# Combined bladder neck preservation and posterior musculofascial reconstruction during robotic assisted radical prostatectomy: effects on early and long term urinary continence recovery

**DOI:** 10.1186/s12894-017-0308-1

**Published:** 2017-12-15

**Authors:** Riccardo Bartoletti, Andrea Mogorovich, Francesco Francesca, Giorgio Pomara, Cesare Selli

**Affiliations:** 1 Urology Unit University of Pisa, Pisa, Italy; 2 Urology Unit AOUP, Pisa, Italy; 3 Urology University Unit, Cisanello Hospital, Via Paradisa 2, 56124 Pisa, Italy

**Keywords:** Prostate cancer surgery, Robot assisted radical prostatectomy, Bladder neck preservation, Posterior musculofascial reconstruction, Urinary continence

## Abstract

**Background:**

To evaluate the effects of combined bladder neck preservation and posterior reconstruction techniques on early and long term urinary continence in patients treated by robotic assisted radical prostatectomy (RARP).

**Methods:**

Two-hundred ninety-two patients who previously underwent radical prostatectomy were retrospectively selected for a case-control study, excluding those with anastomotic strictures and significant perioperative complications and re-called for a medical follow-up visit after their consent to participate the study. They were divided in 3 different groups according to the surgical technique previously received: radical retropubic prostatectomy (RRP) combined with bladder neck preservation (BNP), RARP with bladder neck resection, and RARP combined with BNP and posterior musculofascial reconstruction (PRec).

Functional and oncologic outcomes evaluation were integrated by a questionnaire on urinary continence status, abdominal ultrasound scan, uroflowmetry and post-void urine volume measurement.

Urinary continence definition included the terms “no pad” or “safety pad”.

**Results:**

Two hundred thirty-two patients responded to the phone call interview and were enrolled in the study. They presented comparable age, prostate volume and BMI. Differences in comorbidities, ASA score and medications, did not influence the postoperative functional results, focused on continence outcome.

Early urinary continence was achieved in 49.38% and 24.73% of patients who previously underwent RARP + BNP + PRec and simple RARP respectively (*p* = 0.000)as well as late 12-months urinary continence was obtained in 92.59% and 79.56% of patients.(*p* = 0.01). Late urinary continence in the RRP + BNP group was comparable to the result obtained in the simple RARP group. The potential effects of nerve sparing technique on urinary continence have not been evaluated.

**Conclusions:**

The combined technique of RARP + BNP + PRec seems to be effective to determine early and long term significant effects on urinary continence of patients with comparable body mass index, age and prostate volume. No statistically significant differences were found between the simple RARP and the RRP + BNP groups.

## Background

Postoperative urinary continence recovery after laparoscopic robotic assisted radical prostatectomy (RARP) is related to multiple factors such as patient and disease characteristics, surgical skill and experience and techniques used for surgical demolition and reconstruction.

Previous studies have been conducted by several authors with conflicting results while meta-analysis studies were mainly based on observational retrospective data rather than prospective comparative randomized clinical trials.

Fifty-one articles regarding case series and comparative studies were recently analyzed by Ficarra et al. [1]. Considering the “no pad” or “safety pad” conditions as the continence definition, they found a prevalence of urinary continence after RARP ranging from 89 to 92%. Age, body mass index, lower urinary tract symptoms and prostate volume were the most relevant preoperative predictors of urinary incontinence. The possible role of different surgical techniques was also evaluated in comparative studies. [2–6]. In particular, anterior and posterior reconstructions before the urethro-vesical anastomosis were evaluated in five different studies including 2234 patients. Moreover better early and long term continence rates were found in patients who underwent bladder neck preservation (BNP) and RARP in comparison to those treated by bladder neck resection and reconstruction as also previously reported by other authors [7, 8]. Ficarra et al. concluded that the prevalence of urinary incontinence after RARP is mainly influenced by several factors such as preoperative patient characteristics, surgeon experience but not adjunctive reconstruction techniques. Posterior musculofascial reconstruction seems to offer a slight advantage in terms early continence recovery in comparison with the “no-reconstruction” group. [1].

Walz et al. recently underlined the relevant role of surgical anatomy of the prostate and the adjacent tissues involved in radical prostatectomy, stating that the anatomy varies in each patient, and the approach should be individualized according with cancer and patient characteristics to improve oncologic and functional results at the same time [9].

None of the previous studies evaluated the combination of bladder neck sparing and posterior musculo-fascial reconstruction despite the variability of different parameters related with both the disease and the patient characteristics.

The aim of the present study was to compare the combination of two different surgical techniques such as bladder neck preservation (BNP) and posterior bladder neck reconstruction (PRec) in patients who underwent RARP for prostate cancer treatment.

## Methods

Two-hundred ninety-two patients who previously underwent radical prostatectomy for prostate cancer were enrolled in a retrospective case-control observational study to assess the urinary continence outcomes according the STROBE statement and in accordance with the Declaration of Helsinki. The study was conducted in the same medical center by two different urological teams.

Patients were selected consecutively in order to obtain comparable preoperative predictors of urinary continence such as BMI and prostate volume. Informed consent to participate the study was collected from all the re-called subjects. Patients with anastomotic strictures and significant perioperative complications were excluded.

The patient selection was performed along a period of 7 years from 2009 to 2016. The RRP + BNP procedure was predominantly performed during the first 3 years while the RARP was more frequently adopted during the following 4 years. The surgical procedure choice was determined by the availability of slots for robotics. During the first 3 years the Da Vinci robot was seldom available in a multidisciplinary robotic centre. During the following 4 years it was routinely used despite the number of patients candidate to radical prostatectomy was greater of that included in the robotic slots. The patients have been divided into three different groups. The first included patients treated by RRP according to Walsh [10], BNP and urethro-vesical anastomosis with interrupted sutures. The urinary catheter removal was always done two weeks after the surgery and retrograde cystography control. Urinary continence was investigated 12 months after surgery. The second group included a series of patients who previously underwent RARP (Montsouris technique) and standard urethro-vesical anastomosis according to Van Velthoven [11, 12]. The third group included patients who previously underwent RARP with BNP, posterior urethrovesical reconstruction (PRrec) according to Rocco modified by Coelho and urethrovesical anastomosis according to Van Velthoven [12–14]. The present study includes only patients who did not require bladder neck reduction due to wide caliber (grade 1-2 according to Lee et al.). [15].

The catheter removal was done seven days after the surgery. Continence was retrospectively investigated in the two groups treated with RARP before surgery, immediately after the urethral catheter removal and 3,6 and 12 months respectively.

Patients operated with RARP and standard anastomosis and those treated with open radical retropubic prostatectomy and bladder neck sparing technique represented the two control groups of our retrospective case control study.

All the patients were re-called for a medical follow up visit.

The medical follow up included the evaluation of oncologic and functional outcomes and was integrated with abdominal ultrasound scan, uroflowmetry and post-void urine volume measurement. Moreover a questionnaire regarding the pre and post operative continence was administered as follows:

- Which urinary symptoms did you present prior of the surgery?
- Did you receive other prostate surgeries prior the radical prostatectomy procedure?
- Did you get immediate urinary continence after urinary catheter removal?
- If not, how long did you wait for a satisfactory urinary continence after the radical prostatectomy procedure?
- How many pads do you use in a day?
- Did you receive urethral dilations or surgeries after the urinary catheter removal?

Complete urinary continence definition was described as “no pad” or “safety pad” according with Ficarra et al. [1]. Slight/moderate incontinence has been defined as “no more than 2 pads a day” although this concept may be considered as arbitrary due to the individual perception of “the need to feel oneself clean”. More than 2 pads a day was classified as “severe incontinence”.

Statistical analysis was conducted by comparing the different groups of patients with the Fisher test according the following layout in order to discriminate the potential role of BNP, robotic approach and PRec in the continence recovery in a series of patients with comparable preoperative predictors such as age, BMI and prostate volume between the 3 groups:

- 1) RARP + BNP + PRec vs RARP
- 2) RARP vs RRP + BNP
- 3) RARP + BNP + PRec vs RRP + BNP

Present data are recorded on a dedicated database which includes each single patient clinical but not personal information except for age and BMI. This is the reason because data can’t be shared in a proper repository.

Median values and standard deviations were calculated in each of the series. The power of the study was considered at 80% with a 95% confidence interval.

## Results

The median age and the BMI of patients operated by RRP procedure were 65.7 ± 6.5 years and 27.14 ± 2.40 respectively while that of patients operated by RARP were 69 ± 6.91 years and 26.31 ± 3.19 respectively. (*p* = 0.4). Differences in comorbidities, ASA score and medications, did not influence the postoperative functional results, focused on continence outcome.

Seventy-nine point 45 % of patients responded to the phone recall and received the medical monitoring. All the others (20.55%) were considered as drop out from the study: 6 patients deceased for unrelated diseases and 54 refused to come to the Hospital or receive a telephone interview. The patient selection is described in Table 1.

**Table 1:** Patients selection and patient characteristics

	RARP BNP + PRec. *n (%)*	RARP(no BNP+ no PRec.) *n (%)*	Open RRP + BNP *n(%)*	Total *n(%)*
Selected patients	105	125	62	292
Enrolled patients	81	93	58	232
Age (median) ± SD	69 ± 6.91		65.7 ± 6.5	
BMI Kg/m^2^(median) ± SD	26.31 ± 3.19		27.14 ± 2.40	
Prostate volume				
< 50 cm^3^	4 (5)	18 (19.3)	13 (22.4)	35 (15)
> 50 cm^3^	77 (95)	75 (80.7)	45 (77.6)	197 (85)
Phone call drop out -Pts deceased for unrelated causes	24 (29.6)	32 (34.4)	4 (6.9)	60 (25)
	3	2	1	6
-Pts. who refused to respond the questionnaire or participate the visit	21	30	3	54
*Preoperative clinical data*	*n(%)*	*n(%)*	*n(%)*	*n(%)*
PSA <10 10–20 > 20	69(85.18) 12(14.81) 0 (0)	67(72.04) 23(24.73) 3(3.3)	42 (72.41) 13 (22.41) 3 (5.17)	178 (76.72) 48 (20.68) 6 (2.58)
Gleason score Low risk Intermediate risk High risk	66 (81.48) 11 (13.58) 4 (4.93)	68 (73.11) 18 (19.35) 7 (7.52)	35 (60.34) 17 (29.31) 6 (10.34)	169 (72.84) 46 (19.82) 17 (7.32)
*Postoperative pathological data*	*n(%)*	*n(%)*	*n(%)*	*n(%)*
Stage				
T1-T2 T3	70 (86.41) 11 (13.58)	69 (85.18) 24 (25.80)	47 (81.03) 11 (18.96)	186 (80.17) 46 (19.82)
Gleason score				
Low risk Intermediate risk High risk	39 (48.14) 31 (38.27) 11 (13.58)	41 (44.08) 38 (40.86) 14 (15.05)	24 (41.37) 26 (44.82) 8 (13.79)	104 (44.82) 95 (40.94) 33 (14.22)

*RARP* = robot assisted radical prostatectomy, *BNP* = bladder neck preservation, *PRec*. =posterior reconstruction

Two-hundred thirty-two patients responded the phone recall and participated the medical monitoring outpatient visit.

One-hundred sixty one patients (69.8%) had urinary symptoms prior of the surgery. About 30% of them presented urgency properly treated by anticholinergics. No urinary incontinence was reported. No previous surgeries on the prostate gland have been performed on the 3 series of patients.

Median age and BMI were not statistically different between the groups as well as prostate volume except for the study group (RARP + BNP + PRec) where patients with a smaller prostate volume were less represented than in the two other groups.

Preoperative PSA values and Gleason scores of all patients were mainly included in the low risk group (PSA <10 ng/ml and Gleason score ≤ 6) while overstaging and overgrading were found at pathological analysis in particular in the RARP group. Patient characteristics are described in Table 1. Four experienced urologic surgeons have been involved in the present study. (C.S., A.M., F.F., G.P.). Robotic procedures were initiated in 2007 and by 2009 the learning curve was over. Initially there was limited access to robotics and the great majority of cases were underwent open surgery. Subsequently two additional robots have been acquired and the percentage of robotic procedures increased significantly.

Patients with urgency have been individually evaluated by urine culture with antibiotics administration in case of infection and anticholinergics in case the urine culture did not result as positive. Asymptomatic bacteriuria in uncomplicated patients was not treated by antibiotic therapy.

The mean follow-up time for the RARP and the RRP groups was 26.2 and 51.2 months respectively.

Early continence within 14 days from catheter removal was achieved in 49.38% and 24.73% of patients who previously underwent RARP + BNP + PRec. and RARP respectively (*p* = 0.000). Urinary continence improved progressively in both groups. Late 12- months full continence was obtained in 92.59% of the RARP + BNP + PRec. group and 79.56% of the simple RARP group (*p* = 0.01) (Table 2).

**Table 2:** Urinary continence comparative results between the two groups of patients who underwent to RARP

	RARP BNP + PRec.	RARP(no BNP+ no PRec.)	*p value*
	*n/%*	*n/%*	
Severe incontinence (12 months)	4 (4.93)	14 (15.05)	0.03
Slight/Moderate incontinence (12 months)	2 (2.46)	5 (5.37)	n.s.
Socially acceptable continence (no pads)			
Early	40 (49.38)	23 (24.73)	0.000
At 3 months	63 (77.77)	44 (47.31)	
At 6 months	69 (85.18)	60 (64.51)	
At 12 months	75 (92.59)	74 (79.56)	0.01

Early as well as long term socially acceptable continence was obtained in the patients who underwent to the combined BNP + PRec surgical technique

Similarly complete continence was achieved by 75.86% of patients who previously underwent RRP + BNP and compared to those treated by RARP + BNP + PRec. (p = 0.01) (Table 3). Due to this reason no statistically significant differences were found between the simple RARP and the RRP + BNP groups (Table 4) .

**Table 3:** Urinary continence comparative results between patients who underwent to RARP + BNP + PRec vs those treated by RRP + BNP

	RARP BNP + PRec.	OPEN RRP + BNP	*p value*
	*n/%*	*n/%*	
Severe incontinence (12 months)	4 (4.93)	8 (13.79)	n.s.
Slight/Moderate incontinence (12 months)	2 (2.46)	6 (10.34)	n.s.
Socially acceptable continence (no pads)			
Early	40 (49.38)		
At 3 months	63 (77.77)		
At 6 months	69 (85.18)		
At 12 months	75 (92.59)	44 (75.86)	0.01

**Table 4:** Urinary continence comparative results between patients who underwent to RARP vs those treated by RRP + BNP

	RARP	OPEN RRP + BNP	*p value*
	*n/%*	*n/%*	
Severe incontinence (12 months)	14 (15.05)	8 (13.79)	n.s.
Slight/Moderate incontinence (12 months)	5 (5.37)	6 (10.34)	n.s.
Socially acceptable continence (no pads)			
At 12 months	74 (79.56)	44 (75.86)	n.s.

Severe urinary incontinence was found just in about 5% of patients in the RARP + BNP + PRec. group but in 15% of patients treated with simple RARP despite BMI and patients age were comparable in both groups as well as pathological stage and Gleason score, while prostate volume was greater in the first group.

Adjuvant radiotherapy was performed 13.7%, 30% and 12.3% of patients in the RRP + BNP, RARP, RARP + BNP + PRec groups respectively, at least six months after surgery. The continence status was unaffected by adjuvant radiotherapy in comparison with that observed after surgery.

Patients with moderate/severe incontinence were all counseled and treated by rehabilitation and medical therapy as the first step, then advised to undergo surgical treatments such as sling or artificial urinary sphincter placement.

No patients presented urethral or urethro-vesical anastomosis complications in the RARP groups while 5 (8.6%) patients who underwent to RRP presented strictures of the urethro-vesical junction. Two patients were treated by one step urethral dilation while three underwent trans-urethral incision without further recurrences or complications. These five patients presented long term slight/moderate urinary incontinence.

## Discussion

BMI, prostate volume and patient’s age have been considered as independent predictors of urinary continence recovery by several authors, despite some results seem to be conflictual [16–18].

Comparative continence rates between normal, overweight and obese patients who previously underwent to radical prostatectomy have been recently found by Gozen [19]. Conversely Kumar demonstrated a significantly higher continence rate in a group of patients with none of the risk factors such as age > 70 years, BMI > 35 Kg/m^2^, prostate weight > 80 g. [18]. This is the reason why consecutive patients with comparable risk factors have been selected in the present series in order to exclude possible biases, except for few subjects older than 70 years who demonstrated an increased risk of developing postoperative urinary incontinence.

Konety already provided detailed information regarding the relationship between prostate size and urinary continence after radical prostatectomy, by using the CaPSURE data base which included 2097 evaluable patients. They differentiated 3 different group of patients according to prostate volume (<25, 25–50 and >50 cm^3^.) and found that patients with volume larger than 50 cm^3^ had lower rates of continence. [17]. This is the reason why our patients were classified by discriminating prostate volume less or more than 50 cm^3^.. Patients with lower prostate volume were mainly represented in the simple RARP and the RRP groups despite the group treated by combined RARP and BNP + PRec. demonstrated the best urinary continence rate.

Other relevant variables that have to be considered to obtain the best results in terms of urinary continence include the surgical skill and the surgical technique adopted.

Surgical skill remains one of the most critical issues in urologic surgery and particularly the use of different techniques such as RRP, laparoscopic radical prostatectomy and RARP. All the surgeons participating the study had sufficient experience regarding both the utilized surgical techniques with at least 100 procedures either for RRP or RARP.

The surgical technique adopted may vary in relation with the previously described independent predictors of urinary continence and the disease extension. Other significant parameters such as the prostate gland morphology and the presence of a large third lobe in particular and/or the abnormal anatomy of the prostate apex may also be related with the long term functional results but were not evaluated in the present study [20, 9].

BNP represents a significant step during the surgical demolitive approach to the prostate. The risk of developing surgical positive margins seems to be very low and related to those cancers originated from the gland’s transition zone [21]. None of our patients who received BNP presented positive surgical margins at the prostate base.

Gu et al. recently described the results obtained on a series of 233 patients who previously underwent to RARP with BNP. Early urinary continence with “no pads” was achieved by 69.1% of evaluable patients. [8]. Stolzenburg reported similar results on a retrospective series of 240 patients. [22]. On the other hand, Nyarangi-Dix found significantly higher early and overall continence rates in a randomized controlled trial on 208 patients treated with BNP during radical prostatectomy independently from the open or robotic approach. [21].

Patients treated by RRP + BNP demonstrated comparable results in terms of urinary continence as those who underwent simple RARP. As a consequence the hypothesized advantages of BNP may be substantially undervalued if used in the open RRP approach other than the simple RARP. The robot-assisted technique helps in the surgical field magnification and improvement of anatomical dissection as well as the use of Van Velthoven’s vs interrupted suture. This analysis has been also confirmed by Ficarra that found 11.3% and 7.5% absolute risk of urinary incontinence in patients who previously underwent to RRP and RARP respectively (RARP OR: 1.53; *p* = 0.03). [1]. In our study BNP during RRP likely helped to cover the urinary continence gap between the two surgical techniques.

Similarly the effects of posterior reconstruction introduced by Rocco seems to have more advantages in the early urinary continence recovery other than the long term continence rates. [1, 23]. Urinary continence assessment after reconstruction of the peri-prostatic tissues in patients undergoing RARP was conducted in a controlled trial on a series of 116 consecutive patients prospectively randomized. There was no statistically significant difference in continence recovery between the two groups. [2]. Conversely Hurtes described substantial advantages of the reconstructive group in a series of 72 randomized patients prospectively collected during a period of two years. [5]. The simple RARP group presented an increased number of patients with long term severe incontinence (15.05%) when compared to the combined BNP + PRec. group (4.93%) despite the number of patients with prostate volume lower than 50 cm^3^. was about five times larger in the first of the two groups. This phenomenon may be justified by the larger number of pT3 patients in the simple RARP group (25.8%) in comparison to the other one (13.5%).

The innovative use of the combined technique BNP + PRec. demonstrated significant effects on both the early and the long term urinary continence recovery in comparison with the other two groups. In particular the progressive urinary continence rate recovery between the two groups of patients treated by RARP along a 12 months period, demonstrated an early, mid and long term improvement of continence in favour of the combined BNP/PRec. technique.

To our knowledge this is the first study demonstrating the possible effects of such combined demolitive/reconstructive surgical techniques, although prospective randomized clinical trials should be properly designed to obtain definitive results.

We recognize that the present study has several limitations. This was a retrospective analysis of series of patients who underwent radical prostatectomy along a period of seven years and operated according to different surgical techniques. An objective assessment of baseline continence status was not performed to obtain effective comparable results. The use of pads was arbitrary taken as a proxy for the assessment of postoperative continence status other than the use of validated questionnaires. Finally the effect of nerve sparing technique on urinary continence was not evaluated.

## Conclusions

In conclusion our findings suggest that the combined surgical technique of RARP plus BNP and PRec according to Coelho, provide early and long term statistically significant effect on urinary continence in patients without subjective increased risk of incontinence due to insisting factors such as large prostate volume, high body mass index and age > 70 years.

## Abbreviations

BMI: body mass index
BNP: bladder neck preservation
PRec: posterior musculofascial reconstruction
RARP: robotic assisted radical prostatectomy
RRP: retropubic radical prostatectomy
